# Enhancing Handwriting Performance of Children with Developmental Coordination Disorder (DCD) Using Computerized Visual Feedback

**DOI:** 10.3390/children10091534

**Published:** 2023-09-11

**Authors:** Rachel Bartov, Michael Wagner, Nir Shvalb, Michal Hochhauser

**Affiliations:** 1 Department of Occupational Therapy, Ariel University, Ariel 40700, Israel; rachel.b@orot.ac.il; 2 Department of Special Education, Orot Israel College, Elkana 4481400, Israel; 3 Department of Industrial Engineering and Management, Ariel University, Ariel 40700, Israel; wagnerm@ariel.ac.il; 4 Department of Mechanical Engineering and Mechatronics, Ariel University, Ariel 40700, Israel; nirsh@ariel.ac.il

**Keywords:** handwriting, intervention, pressure, regulation, spatial, temporal, visual feedback

## Abstract

Recent studies have analyzed the writing metrics of children with developmental coordination disorder (DCD) using computerized systems. To date, the use of computerized visual feedback to improve handwriting has not been investigated. This study aimed to examine the effects of computerized visual feedback on handwriting performance in time, spatial orientation, and pressure indices for children with DCD. Twenty-seven children aged 7 to 12 years with DCD assessed by the Movement Assessment Battery for Children and the Developmental Coordination Disorder Questionnaire received one weekly intervention session for 8 weeks, during which they twice copied an excerpt onto a tablet. Once, they received visual feedback where the writing color corresponded to the degree of pressure on the writing surface, and once they received no visual feedback. The two conditions were counterbalanced throughout the sessions. Pre-intervention sessions were compared with post-intervention sessions and with new texts for time, spatial orientation, and pressure measures. The findings revealed significantly decreased total and mean letter writing, in-air, and writing time and increased capacity in the visual feedback condition. In the spatial variables, a significant decrease in letter height variance was found. Pressure increased significantly throughout the intervention with visual feedback, whereas it decreased post-test in the writing task in both conditions and was maintained in the new text. Visual feedback intervention can increase the kinesthetic–haptic feedback required to regulate pressure during writing, promoting more efficient feedforward processes and improving output quality and capacity. The training effectiveness was transferable, and the intervention accessibility could increase student autonomy.

## 1. Introduction

Writing is a unique, human, cultural asset essential for academic functioning in school. The manual writing process fosters a wide range of cognitive functions and neural connections. It facilitates the acquisition of reading skills and language performance, contributing to children’s future academic learning abilities [1,2,3,4,5].

Children with developmental coordination disorder (DCD) are defined in the Diagnostic and Statistical Manual of Mental Disorders [6] as exhibiting difficulties in coordinating gross and fine motor skills relative to expectations for their chronological age. These difficulties, such as in writing, may affect their daily functioning and academic performance.

Research focusing on writing difficulties in children with DCD found that over 80% of them experience challenges in writing compared to typically developing children [7,8,9]. These challenges include slow writing and illegibility [10,11,12,13]. There are various methods for acquiring writing skills and treating children who struggle with writing, including those within the population of children with DCD (e.g., the Cognitive Orientation to Daily Occupational Performance program [14] and neuromotor task training [15]). The goal of these intervention methods is to promote optimal writing acquisition. By emphasizing legibility and writing speed measures, they enable future high-level learning and writing skills [11,13,16,17,18,19,20,21].

Handwriting intervention for children with DCD is based on sensory–motor learning ability. Researchers have emphasized the importance of practicing motor control and self-monitoring to improve motor performance [16,22,23,24]. Training pressure control using visual feedback has been shown to enhance the child’s integration of perceptual and functional motor components, which is crucial for handwriting performance [25,26]. Different approaches to using visual information for children with DCD exist. One approach suggests that children with DCD rely more on “online” visual feedback (rather than preplanning a strategy before the movement) for production and movement control and, thus, require additional processing time [27]. Others argue that these children struggle with planning attentional focus, judgment, and synchronization, making visual feedback insufficient to address their difficulties [28]. 

Writing requires spatial–visual coordination involving size and shape components beyond simple visual–motor coordination, which increases the complexity for children with DCD [29]. Neurologically, when a motor plan is required, the brain’s motor cortex sends instructions for action through the motor pathways. Simultaneously, the brain networks receive information and connect to the cerebellum and prefrontal cortical regions. Thus, the network activity allows dynamic movement correction and adaptation through real-time sensorimotor feedback during action [17,30]. This mechanism highlights the significance of feedback preceding action (feedforward), which develops slower than post-action sensorimotor feedback. Feedback preceding action allows correcting and adjusting movements during the complex action of writing. Findings in the field of motor learning have indicated that effective feedback for children’s learning is external feedback provided during action. External feedback allows for adjustments and precision of movement during execution, contributing to improved feedback for subsequent actions. Various studies have shown that visual feedback is the most effective and optimal feedback for children’s learning [31]. 

So far, digital tools to support children with DCD have been utilized mainly for handwriting assessment, not intervention. The one exception, Chang and Yu ’s 2017 study [25], combined tactile–haptic systems with visual perception training. It found improvements in writing speed and accuracy but not in letter formation. Therefore, the main goal of our study aimed to enhance handwriting performance in children with DCD using an innovative visual feedback digital tool. 

We hypothesized that, post-intervention, there would be decreases in the time needed to copy a written excerpt, the average one-letter writing time, writing time (without in-air time), and in-air time (i.e., pausing the hand in the air while writing), and an increase in writing capacity (the ratio of the number of written letters to time) [13,32,33]. Second, we hypothesized that decreases would be found in the average letter height and width and their variances, in letter and word spacing and their variances, in letter and word area, and in the number of erasures, and that increases would be found in the percentage of legible letters (spatial) [10,19,25,32,33]. Alongside the research, the prevailing clinical approach has been that children with DCD exert higher pressure when writing [9,34] than children with typical development and consequently become fatigued [8]. However, some studies demonstrated contrasting evidence—that children with DCD exert lower or no different pressure than typically developing children [33]. Considering the evidence, we hypothesized that the writing pressure would decrease post-intervention [35,36,37]. Furthermore, we expected that the same pressure level would be maintained throughout the writing task. Thus, our third hypothesis was that we would see a decrease in the overall pressure on the writing surface [35,36], and the fourth hypothesis was that we would see an enhancement in the ability to maintain consistent pressure on the surface during the writing task [37,38]. Finally, we believed these enhancements would be retained and transferred to writing a new text after the final intervention session compared to the initial pre-intervention session [16,29,39,40]. 

## 2. Materials and Methods

### 2.1. Participants

Twenty-seven children aged 7 to 12 years who had participated in the first phase of this cross-sectional study composed the sample for this second-phase study. The first phase was a comparison study to assess manual and computerized tools. The inclusion criterion for both phases was students identified with DCD (≤5%) or suspected DCD (≤6–15%). Due to the lack of sufficient diagnostic examination for DCD in school-aged children by child neurologists or developmental physicians, this criterion was based on standardized testing by an occupational therapist and a parental questionnaire to identify children with a high probability of DCD (pDCD). They were assessed as with DCD by the Movement Assessment Battery for Children (MABC-2 [34] and the Developmental Coordination Disorder Questionnaire (DCDQ [41]) with cutoff scores by age (7.00–7.11 years, DCDQ ≤ 46; 8.00–9.11 years, DCDQ ≤ 55; 10.00–12.00 years, DCDQ ≤ 57). Table 1 displays other relevant characteristics.

### 2.2. Research Tools

#### 2.2.1. Movement Assessment Battery for Children

The MABC-2 [41] is a standardized, valid test to identify children with DCD or pDCD between 3 and 16 years old. We used the appropriate battery for ages 7.00 to 10.11 years. Each battery includes eight motor tasks divided into three subdomains: manual dexterity, ball skills, and balance. Scores between 6% and 15% indicate at-risk for motor impairment; scores lower than 5% indicate the presence of a motor disorder.

#### 2.2.2. Developmental Coordination Disorder Questionnaire

The DCDQ [42] is a validated questionnaire for parents of children between 5 to 15 years who may be at risk for DCD. Parents evaluate their child’s daily motor performance on 15 items in three areas—movement control, fine motor skills and handwriting, and general coordination—on a scale from 1 (does not describe my child at all) to 5 (completely describes my child).

#### 2.2.3. Handwriting on a Tablet

A Wacom Cintiq computerized tablet was used as a digital writing surface. The tablet features a 14-line background mimicking the layout of the children’s notebooks. It was covered with a screen protector to increase friction, similar to paper. The tablet was positioned about 2 cm from the edge of the table, and the children wrote with a stylus similar to a regular pen (using Pen Painter^®^ software, 14.24.28127.4 (MFC Version, 2020). They copied a writing section from the Hebrew Handwriting Evaluation [43]. The data (x, y, time, and pressure) were obtained at a sampling rate of 133 kHz.

**Figure 1:**
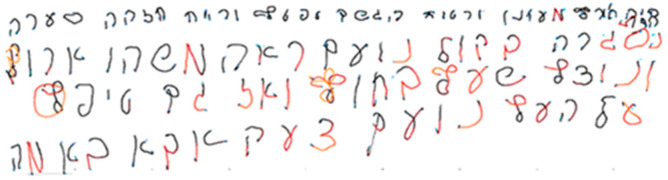
Example of the writing color change according to the degree of pressure on the tablet in an intervention session. Note: Red indicates high pressure on the tablet surface, whereas black indicates appropriate pressure.

The writing color changed according to the pressure the child applied on the surface while writing, providing immediate visual feedback. High pressure resulted in red text, low pressure in blue text, and moderate pressure corresponded to black text on a white background (see Table S1 and Video S1 in Supplementary Materials), similar to pencil writing in a notebook (Figure 1). The colors were calibrated based on average pressure levels among the population of typically developing children, as determined in the initial research stage. Figure S1 in the Supplementary Materials provides the conversion of pressure levels determined in the Wacom tablet version to Newtons.

### 2.3. Procedure

Parents of the study participants signed informed consent forms and completed the DCDQ [42]. The researcher, a trained occupational therapist, administered the test batteries (MACB-2, Hebrew Handwriting Evaluation, and tablet-writing task) to each participant individually in a quiet room with an appropriately sized table and chair. Each training session lasted approximately 20 min.

The students were informed that they would practice writing on the tablet for a few minutes every week for 8 weeks. Before the practice, they had a brief trial in tracing a line of five shapes for 20 s each to experience the principle of color feedback corresponding to pressure. The shapes included non-letter shapes with straight and curved lines resembling Hebrew letters. Subsequently, we explained to the children that they would copy a writing excerpt twice: once with visual feedback and once without color change (black on white). The students copied the full writing excerpt (47 words) from a handwriting assessment or copied it for 5 min (whichever they completed first).

**Figure 2:**
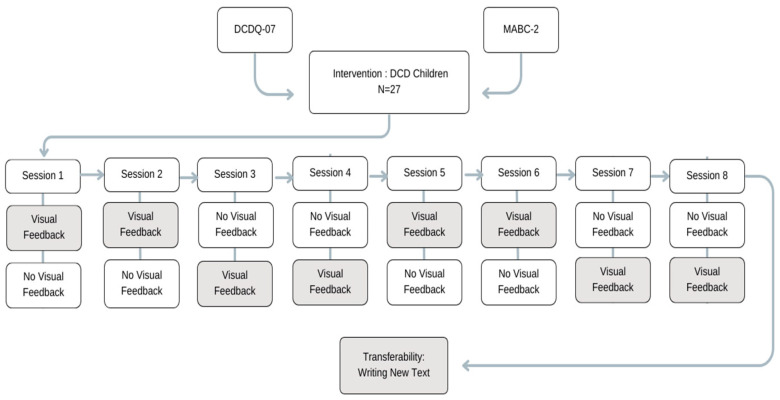
Study design demonstrating the flow of randomly counterbalanced feedback conditions throughout the sessions.

A within-subject design was conducted between copying the writing excerpt under the two conditions—with and without visual feedback—randomly counterbalanced throughout the sessions. In each session, the participants wrote with and without visual feedback (Figure 2). 

The cross-sectional design throughout the study resolved the possibility that developmental factors might interfere during a withdrawal intervention period. Comparing the pre-intervention to post-intervention sessions under different conditions (i.e., with and without visual feedback) allowed us to assess whether there was a learning process related to the with visual feedback condition. Additionally, comparing the pre-intervention writing performance with the eighth (final) session—in which the children copied an additional new excerpt—enabled the examination of transferability (i.e., acquired skills retention after the intervention period).

### 2.4. Statistical Analyses

The data were processed using MATLAB^®^ to calculate the temporal and spatial measures and analyzed with IBM SPSS (Version 27). The participants’ demographic variables were described using descriptive statistics, frequencies, percentages, means (*M*), and standard deviations (*SD*). A series of two-way analysis of variance (ANOVA) tests with repeated measures were conducted with Bonferroni corrections to compare writing performance across temporal, spatial, and pressure variables. These compared performances at the pre-intervention (first) session to performance at the post-training (eighth) session while also comparing performance with visual feedback to performance without visual feedback—four repeated measures: 2 pre-/post-intervention × 2 intervention conditions (with/without visual feedback). Post hoc analysis using *t* tests were conducted to examine the source of interaction when normal distribution was assumed (Levene’s test *p* > 0.05). Additionally, Cohen’s d effect size was calculated. Subsequently, the performance in writing a new text after the post-intervention session was compared to writing the text in the first and eighth training sessions in writing a new text. 

We conducted a series of two-way ANOVAs with repeated measures and Bonferroni corrections and *t* tests for the total segment differences to compare the pressure throughout the writing task (divided into five segments). The differences between the five writing segments (within variables) were examined, as was the presence of an interaction effect between the with and without visual feedback conditions. 

## 3. Results

### 3.1. Temporal Measures

Significant differences were found between the pre- and post-intervention results. The findings show that post-intervention, the time to copy the whole section, the one-letter writing time, the writing time, and the in-air time decreased. Additionally, the capacity (i.e., the number of letters relative to the writing time) increased post-intervention (Table S2 in Supplementary Materials).

**Figure 3:**
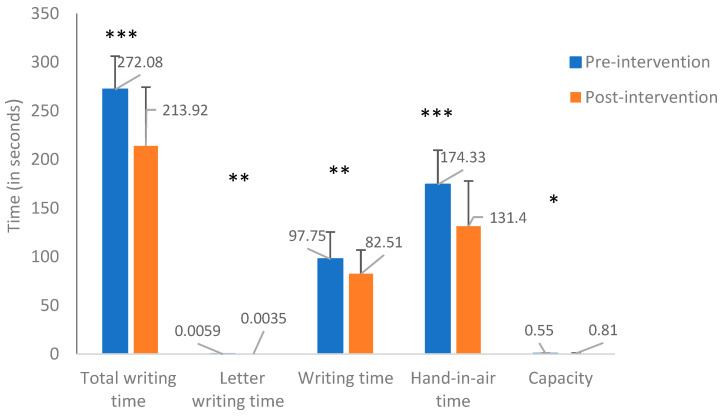
Pre- and post-intervention temporal measures of writing with visual feedback. * *p* < 0.05; ** *p* < 0.01; *** *p* < 0.001.

**Figure 4:**
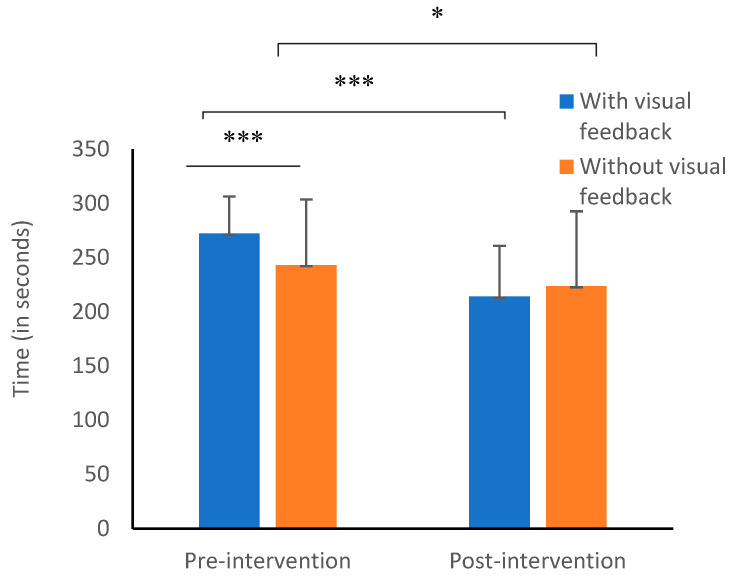
Interaction effect between the total writing time (pre- and post-intervention) X group (with and without visual feedback). * *p* < 0.05; *** *p* < 0.001.

A main effect was found for the total writing time (*p* = 0.001), such that writing was significantly shorter post-intervention. In addition, a significant difference was found between the intervention conditions (with or without visual feedback), *F*(1,26) = 4.74, *p =* 0.04, ղ² = 0.154. The total writing time with visual feedback (Figure 3) was slightly longer than without visual feedback (Figure S2). In addition, a significant interaction was found between time (pre- and post-intervention) and the intervention condition (with and without visual feedback), *F*(1,26) = 22.29, *p* = 0.000, χ² = 0.462 (Figure 4).

Follow-up analyses (*t* tests) were conducted to examine the source of the interaction. Examining the differences at pre-intervention indicated significantly longer writing time when the children received visual feedback (*M* = 272.08, *SD* = 34.28), compared to the performance duration without visual feedback (*M* = 243.06, *SD* = 46.84), *t*(26) = 5.22, *p* = 0.000, Cohen’s *d* = 0.70. 

On the other hand, at the end of the intervention period, the performance duration, both with visual feedback (*M* = 213.92, *SD* = 60.54) and without visual feedback (*M* = 223.56, *SD* = 69.05, decreased— *t*(26) = 1.48, *p* = 0.15. No significant differences were found between the writing with visual feedback and without visual feedback. However, the pre-intervention to post-intervention change seen with the visual feedback was significantly more prominent, *t*(26) = 6.69, *p* = 0.001, with a large effect size, Cohen’s *d* =1.18, than the pre- and post-performance difference in the without visual feedback condition, *t*(26) = 2.39, *p* = 0.24. 

After examining the overall time measurements, we analyzed additional temporal variables. When comparing the time to write one letter, writing time, and writing capacity (number of letters written in the total writing time) pre-intervention to post-intervention, we found a significant main effect for time (pre-/post-intervention), a significant effect for intervention condition (with/without visual feedback), and a significant interaction effect of time X intervention condition. Additionally, there were significant main and interaction effects for in-air time without a significant effect for intervention conditions. (The findings are presented in the Supplementary Materials.)

### 3.2. Spatial Measures

**Figure 5:**
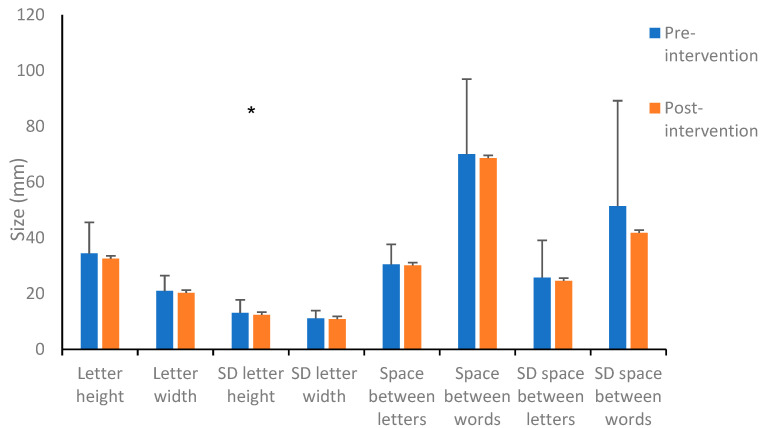
Pre- and post-intervention spatial measures of writing with visual feedback. * *p* < 0.05.

The standard deviation of the mean height of a letter significantly decreased post-intervention compared to pre-intervention, *p* = 0.003 (Figure 5). Additionally, an interaction effect was found in the visual feedback condition, *F*(1,26) = 4.67, *p* = 0.04, ɳ² = 0.153 (illustrated in Figure S7 and Table S2 in Supplementary Materials).

**Figure 6:**
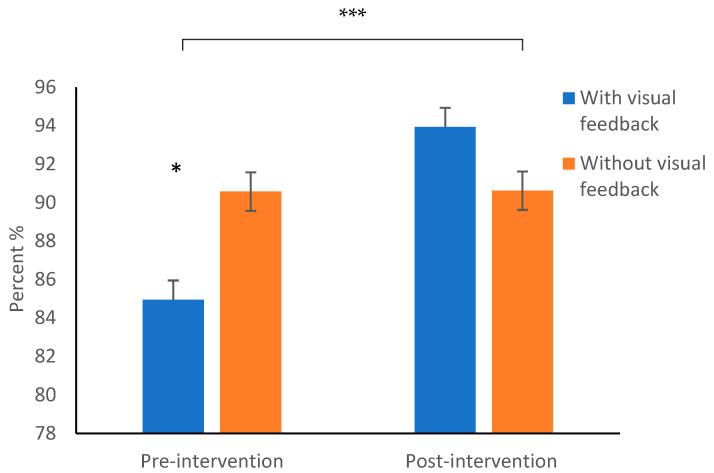
Differences in the number of (non-omitted) letters written. * *p* < 0.05; *** *p* < 0.001.

There was a significant increase in the standard deviation of the space between letters, *p* = 0.03, and between words, *p* = 0.02, in the without visual feedback condition (Figure S7). We conducted a repeated measures ANOVA using a Bonferroni correction to test the hypothesis that there would be an improvement in the percentage of letters written (i.e., non-omitted letters) post-intervention compared to pre-intervention. We found a significant main effect of time (pre-/post-intervention), *F*(1,26) = 8.13, *p* = 0.008, ղ² = 0.238 (Figure 6). In contrast, no significant main effect for visual feedback condition was found. However, there was an interaction effect, *F*(1,26) = 11.45, *p* = 0.002, η² = 0.306, as shown in Figure S9.

Post hoc analyses *(t* tests) were conducted to examine the source of the interaction. The findings indicated that the mean percentage of letters written post-intervention significantly increased compared to pre-intervention in the visual feedback condition, *t*(26) = 3.35, *p* = 0.002, Cohen’s *d* = 0.61. However, the percentage of letters written pre- and post-intervention without visual feedback did not yield a significant difference.

Further, no significant changes were found post-intervention compared to pre-intervention in measures of average height and width of letter writing, letter area and word area, spaces between letters and between words, and the number of erasures throughout the writing task. (For additional results for spatial variables, see the Supplementary Materials).

### 3.3. Pressure Measures

**Figure 7:**
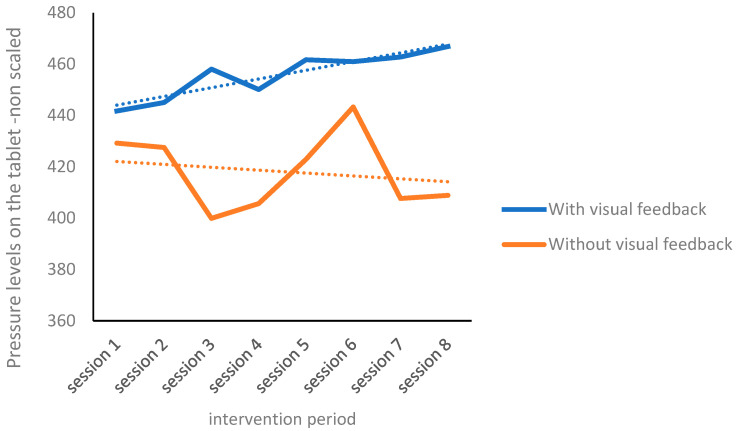
Writing pressure pre-intervention (first session) and post-intervention (eight session) with and without visual feedback.

There was no significant main effect of time (pre-/post-intervention); however, we found a significant main effect of visual feedback, *F*(1,26) = 15.76, *p* = 0.001, η² = 0.38, and an interaction effect, *F*(1,26) = 13.17, *p* = 0.001, η² = 0.34. Post hoc analyses were conducted to examine the source of the interaction. Contrary to our expectations, the overall writing pressure significantly increased between pre-intervention (*M* = 440.27, *SD* = 65.43) and post-intervention (*M* = 467.28, *SD* = 46.31), *t*(26) = 2.67, *p* = 0.013, in the visual feedback condition (Figure 7). The change was moderate with a Cohen’s *d* effect size of 0.5. In contrast, we found no significant change in the without visual feedback condition.

The findings indicated no significant differences in the overall writing pressure between with and without visual feedback conditions at pre-intervention. However, a significant post-intervention difference was found between the overall writing pressure in the with visual feedback (*M* = 467.28, *SD* = 46.31) and without visual feedback (*M* = 408.18, *SD* = 80.18) conditions, *t(*26*)* = 5.16, *p* ≤ 0.001, with a large effect size, Cohen’s *d =* 0.90. Additionally, a comparison was made to examine differences in the writing pressure across all eight intervention sessions (Table S3 in Supplementary Materials). 

**Figure 8:**
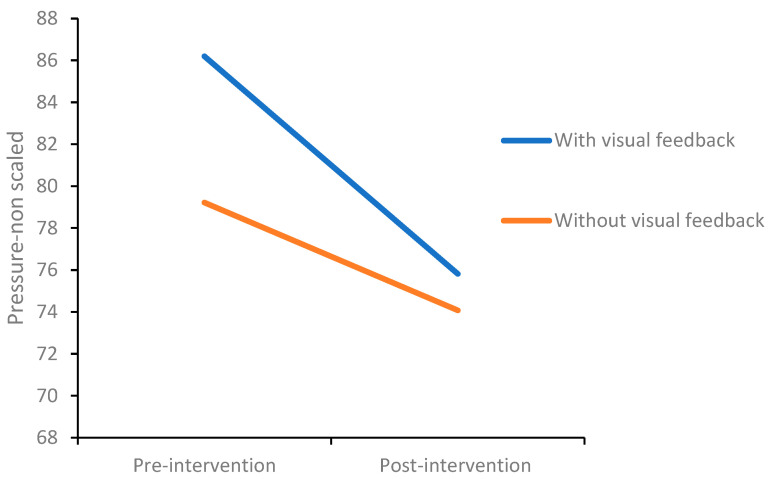
Writing pressure pre-intervention (first session) and post-intervention (eighth session), with and without visual feedback.

Upon examining the standard deviations of the mean writing pressure (Table S2 in Supplementary Materials), the overall standard deviations significantly decreased post-intervention in both the with and without visual feedback conditions (Figure 8). A significant main effect of time (pre- and post-intervention) was found, *F*(1,26) = 12.57, *p* = 0.002, η² = 0.32. No significant effect was found for the intervention condition.

To examine whether the children maintained the same level of pressure throughout the writing task, we divided the writing task into five equal segments (by the number of letters the child wrote in the passage). There was a significant increase in pressure from pre- to post-intervention with visual feedback but no significant differences without visual feedback. Additionally, significant differences between pre-intervention segments were observed in the visual feedback condition. Table S4 and Figures S10 and S11 in the Supplementary Materials present the writing pressure at pre- and post-intervention.

### 3.4. Transferability

To test the hypothesis that there would be transferability of the improvements in time, space, and pressure measures after the final intervention session, we asked the children to write a new text after the intervention period. We conducted ANOVA repeated measures analyses with Bonferroni corrections comparing pre-intervention performance measures with and without visual feedback to writing the new text post-intervention. The findings revealed a significant decrease in time when writing a new text after the 8-week intervention period compared to the time writing the pre-intervention text, *p* = 0.001. The improvement was significant in writing with visual feedback versus writing without visual feedback: In the initial session, writing with visual feedback took significantly longer than writing without visual feedback. However, when writing the new text post-intervention, the writing time with visual feedback was shorter, with a large effect size, Cohen’s *d* = 0.90, than the pre-intervention writing time without visual feedback, Cohen’s *d* = 0.26 (Figure S12 and Table S2 in the Supplementary Materials present the detailed results).

Comparing the new text’s spatial measures from pre- to post-intervention revealed a significant difference in the standard deviation of letter spacing between the pre-intervention without visual feedback condition (*M* = 22.54, *SD* = 8.06) and the post-intervention writing of a new text (*M* = 28.51, *SD* = 9.37), *p* = 0.01. Additionally, fewer letters were omitted post-intervention when writing the new text in the visual feedback condition. There was a significant difference in the percentage of letters written in the visual feedback condition pre-intervention (*M* = 84.80, *SD* = 18.76) and the new text post-intervention (*M* = 90.19, *SD* = 10.71), *p* = 0.003, but no significant difference was found in the without visual feedback condition (*M* = 90.26, *SD* = 13.48). Further, no significant differences were found between pre-intervention and the post-intervention new text in the other spatial measures: the letter height and width, standard deviation of letter height and width, spacing between letters and words, standard deviation of word spacing, average area of a letter and word, and number of erasures throughout the writing task. 

The findings revealed no significant differences between the pre-intervention writing pressure in both conditions to the mean writing pressure of the post-intervention new text (see Table S2 in the Supplementary Materials). A significant increase in writing pressure was found without visual feedback between pre-intervention (*M* = 423.30, *SD* = 92.45) and writing a new text post-intervention (*M* = 470.84, *SD* = 47.06), *p* = 0.006. Similarly, a significant increase was found in the visual feedback condition between pre-intervention (*M* = 441.44, *SD* = 66.43) and the post-intervention new text, *p* = 0.05. Nevertheless, significant differences in standard deviations were seen only between the pre-intervention in the visual feedback condition (*M* = 86.37, *SD* = 14.07) and in writing the new text post-intervention (*M* = 76.43, *SD* = 11.71), *p* = 0.001, indicating a decrease in the pressure variance when writing the new text post-intervention.

Comparisons of the writing pressure among the five segments of the new text and between the new segments and pre-intervention in the without visual feedback condition (matching each segment to its parallel segment) are provided in the Supplementary Materials.

When comparing the writing pressure in the new text versus the writing pressure in the final intervention session in the without visual feedback condition, we found significant differences in all five segments—respectively, *t*(25) = 3.45, *p* = 0.003, *t*(25) = 5.89, *p* = 0.000, *t*(25) = 4.73, *p* = 0.000, *t*(25) = 4.92, *p* = 0.000, and *t*(25) = 4.76, *p* = 0.000. In contrast, there were no significant differences in any segment of the visual feedback condition, emphasizing the global retention of the handwriting improvements achieved.

## 4. Discussion

This intervention experiment aimed to test the effects of computerized visual feedback on handwriting performance in time, space, and surface pressure measures for children with DCD. The results showed significant improvements in the children’s writing performance from the pre-intervention session to post-intervention in various time measures (total time, one-letter writing time, in-air time, writing time, and writing capacity). Furthermore, the decrease in the time to write one letter, writing time, and in-air time due to the intervention with visual feedback was significant compared to writing without visual feedback. These temporal improvements were also evident in writing a new text after the intervention period. The spatial measures showed improved uniformity and letter formation, demonstrated by a decrease in the variance of letter height in writing with visual feedback and an increase in the spacing between letters and words in writing without visual feedback. Additionally, the percentage of written letters increased after the intervention, with fewer letter omissions. Improvements were also seen in writing the new text after the intervention period, with increased visual spacing variance without visual feedback and a higher percentage of correctly written letters with visual feedback. Further, the results showed that the pressure on the surface decreased slightly in performance without visual feedback, but significantly in performance with visual feedback. The improved pressure uniformity following the intervention was reflected in decreased post-intervention pressure variance, indicating more homogeneous pressure activation with visual feedback. This improvement was also retained in writing a new text. 

These findings reinforce that the learning process during the intervention period was preserved and transferred into the new text—which was not part of the intervention. When comparing the five segments of the new text to the corresponding five segments of the eighth session, no significant differences were found, indicating performance preservation even in the transfer to a new text. No differences were found between pre- and post-intervention performance in spatial measures, such as letter height and width, spacing between letters and words, letter and word area, and number of erasures in the segment, and no decrease in pressure on the surface was seen following the intervention.

### 4.1. Temporal (Time) Measures

The most prominent impact of the intervention was observed in the improved temporal measures and writing efficiency, which is highly significant in the daily functioning of children with DCD. Greater writing efficiency and less time may contribute to improving their academic performance and integrating them into classroom learning.

The clear improvement in time measures following intervention with visual feedback supports the argument that children with DCD have the ability for motor learning [17,26]. Motor learning refers to a change in behavior and skill acquisition associated with training or practice [26]. Children with DCD exhibit a wide range of difficulties acquiring new motor skills. Some show adaptation and performance coping abilities, whereas others exhibit difficulties and deficits [16,39]. This study’s significant improvements in time measures indicate that children with DCD can improve their writing pace through intervention. They demonstrated task adaptation and better performance coping in the temporal dimension. In addition, it seems that color feedback serves as an intuitive and immediate effective feedback mechanism with the potential to bring about significant improvement. However, it is important to consider the natural learning process and adaptation to the feedback structure during practice, as evident in similar studies focused on the process of new acquisition [4,44].

At the beginning of the intervention, the performance duration was longer with than without visual feedback. This can be explained by the fact that the beginning of the intervention involved learning the new task of writing with visual feedback in the form of color change, which needs a learning and adaptation process. This process requires closed-loop feedback control based on visual feedback instead of an automatic process characterized by faster feedforward monitoring [4,39]. From this study’s findings, it appears that—in adapting to a new task of writing on a tablet with visual feedback—the children need more execution time at the beginning of the process. Later, however, their performance rate improved significantly. By the end of the intervention period, the writing duration had decreased significantly in the performance with visual feedback, even to values lower than those without visual feedback.

Furthermore, a neurophysiological explanation can be suggested. Children with DCD may have different activity in the cerebellum, affecting their motor learning [24]. In sensorimotor-based learning, particularly with visual feedback, the rate of learning and changes following new task acquisition occur differently. Initially, it takes much more time to perform the new task. However, following the intervention with visual feedback, learning ability seems to lead to noticeable improvement [24,45]. 

Research has suggested that this process occurs through the activation of a mirror neuron system mechanism. The mirror neuron system hypothesis posits that mirror neurons, activated both during an individual’s action and when observing a similar action in others, create a bridge between perception and action, aiding in action comprehension and imitation. This concept has significant implications for social cognition and skill acquisition through observational learning when an individual sees or imagines a motor action [46]. This process is also based on mental imagery following the experience, enhancing performance and feedback processes for action (feedforwards), which contribute to faster and more efficient execution. Compared to typically developing children, the mirror neuron system function among children with DCD is reduced and more complex when performing writing activities that involve motor execution combined with the cognitive and extra-cognitive components of language, planning, and visuospatial skills [30,47]. However, the post-intervention improvement observed among the children with DCD in this study demonstrates their capacity to enhance their performance with appropriate intervention. 

Another compatible explanation is based on Gibson’s Direct Perception Theory [48,49,50], also referred to as the “ecological approach”. This theory emphasizes the active role of the perceiver and the importance of the environment in shaping perception. It suggests that perception is not solely based on the internal processing of sensory information but is also influenced by the direct interaction between the perceiver and the surrounding environment. This theory focuses on how organisms perceive affordances, which are the action possibilities that the environment offers to an individual. According to Gibson’s theory, perception and action are tightly coupled. The theory suggests that the information directly available in the environment guides an individual’s actions. In the case of children with DCD, who struggle with motor coordination, there may be disruptions in the typical perception–action coupling. Their ability to accurately perceive relevant environmental cues for guiding actions might be compromised, leading to difficulties in planning and executing movements [51]. In this view, the affordances of visual feedback provide an opportunity for action whereas the condition of no visual feedback requires a lengthier process of reconstructing a representation of the letters. 

### 4.2. Spatial Measures

The findings showed no significant changes in most measured spatial dimensions: the height and width of a letter, letter and word spacing, letter and word areas, and number of erasures in the written excerpt. However, the intervention with visual feedback reduced the variance in letter height. Moreover, increased standard deviations of letter and word spacing and letter height were observed in the without visual feedback condition. In other words, it is more difficult to maintain the uniformity of the writing without visual feedback. Additional studies indicating that children with DCD have difficulties in the visual–spatial processing domain [40,52] support these findings. Multiple spatial variables in writing affect the final product. The letter design, letter size, letter and word spacing, and missing or excessive letters are all part of these components, and there is significant variance in their execution among children with DCD. The wide range of relationships between these components affects the children’s handwriting legibility and the quality of the writing product [7]. 

It can be argued that no significant improvement in most spatial measures was seen because spatial aspects, such as size, spacing, and alignment, are also influenced by motor adaptation during task execution [53]. In this study, the children had to perform a complex writing task while simultaneously focusing on the various writing components (spelling, visual–motor coordination, letter size, etc.) and the visual feedback component during action. In contrast to practicing a simple motor activity, this demanding task combines simultaneous motor activity and perceptual–cognitive components (tracking the content being written, spelling processes, sequence, and control) [30]. Thus, the children may have had difficulty improving the simultaneous integration of specific spatial components.

In the spatial perception field, the importance lies not only in the variables, such as size or spacing, and their influence on the quality and legibility of the writing but also in maintaining consistency across the writing task. A high degree of writing variance decreases clarity and requires more energy and time to interpret the text. Children with DCD struggle to maintain consistency in their writing, instead highly varying the width, height, area, spacing, and erasures. In our study, this variance persisted in both conditions, continuing to characterize the participants even throughout the intervention sessions. Despite the intervention, there was no significant difference in letter height and width or in letter and word spacing between the first and eighth sessions. 

However, it is interesting that the intervention with visual feedback contributed to a significant reduction in letter height variance; post-intervention, there was much greater homogeneity. In Prunty et al.’s [37] study, the researchers found a greater variance in writing among children with DCD compared to children with typical development. These authors noted that resizing, spacing, writing on the line, and more complex letter design require more effort for children with DCD, resulting in greater variance. Our findings indicate that, without visual feedback, the variance increased significantly (standard deviation of space between letters and between words). In addition, in the with visual feedback condition, the letter height standard deviation decreased. The results provide vital evidence to support that intervention with visual feedback has the potential to affect the spatial component of writing. It seems that performance without visual feedback leads to increased variance, and performance with visual feedback assists in maintaining consistency and reducing variance. 

However, due to the high heterogeneity among children with DCD in performance overall and spatial measurements in particular and because the magnitude of differences can vary widely, it is necessary to approach writing performance in a multidimensional manner (along with time and other variables) rather than focusing on a single set of characteristics, such as spatial variables [53]. Furthermore, the fact that there were few significant changes in spatial measurements between pre- and post-intervention writing strengthens the change effects observed in the temporal measures. These findings emphasize that the temporal improvement does not come at the expense of a decline in the quality of writing performance in spatial measures. The fact that only a few spatial measurements improved contrary to the hypothesis can be explained by Fitts’ law, where there is a trade-off between movement duration and precision, which may affect the product quality [54]. 

Another interesting finding is that the total percentage of letters written increased in the intervention with visual feedback. The findings of Coradinho et al. [55] findings that children with a higher average absolute velocity wrote a larger number of characters support our results.

### 4.3. Pressure Measures

The hypotheses on the pressure exerted on the writing surface were partially confirmed. Comparing the intervention with visual feedback to the intervention without visual feedback indicates that—in both conditions—children with DCD increased their writing pressure during the writing task. As evident in the overall mean pressure and the pressure measurements when the writing task was divided into five segments, this finding is contrary to the hypothesis that training would lead to a decrease in pressure.

In analyzing the five segments, we observed a general tendency to increase pressure (pre-intervention with visual feedback, there was a significant difference between Segment 1 and Segments 3–5; post-intervention with visual feedback, a significant difference was found in the performance in Segments 1–3). We found that post-intervention, there were fewer fluctuations in pressure during the writing task (from Segment 1 to Segment 5) in both intervention conditions (with or without visual feedback). Because the mean pressure was higher with than without visual feedback, it appears that intervention contributed to a more homogeneous performance.

Additionally, a decrease in pressure variance was observed post-intervention in both intervention conditions, indicating that intervention supports more homogeneous performance. These findings indicate that, although the hypothesis of a decrease in writing pressure was not confirmed following the intervention sessions, the writing pressure was modulated throughout the writing task.

The fact that children with DCD increased pressure on the writing surface during the intervention sessions with visual feedback can be explained by the complex nature of the writing task. It requires cognitive, linguistic, and motor resources in addition to the proprioceptive–kinesthetic component required for pressure modulation. The simultaneous complexity of these processes possibly made it difficult for children with DCD to perform fine motor control during writing, leading them to continue exerting increased pressure throughout the intervention sessions to enhance the proprioceptive–kinesthetic feedback received during writing. According to Wilson et al. [24], children with DCD rely heavily on feedback processes because they experience “neural noise” in their sensory–motor system. Other scholars argued that due to this “neural noise”, the children struggle to rely on feedback mechanisms to correct errors, leading to an increased need for sensory stimulation. They attempt to modulate and respond accordingly only when the discrepancy is large enough [9]. This “neural noise” could potentially explain the significant difference observed in writing pressure between the beginning of the writing task and the last three segments during pre-intervention execution with visual feedback and between Segments 1 to 3 at post-intervention execution compared to the execution without visual feedback, where no significant differences were observed in either period. The visual feedback experience appears to have led the children to increase the proprioceptive–kinesthetic pressure, particularly at the beginning of the writing task. Additional support is provided by Wulf’s motor learning theory. This theory is grounded in the idea that an external focus facilitates automatic and natural movement execution by allowing the motor system to self-organize, whereas an internal focus can lead to conscious interference and reduced performance quality [56]. According to this theory, visual feedback constitutes an external focus that encourages action, compared to performance without visual feedback that relies only on the internal focus resulting in fewer changes. On the other hand, other studies have shown a tendency of children with DCD to exert less pressure on the surface than children with typical development. According to this approach, the increase in pressure during the intervention period may reflect the children’s efforts to function more like their typically developing peers

In contrast to the findings from the intervention with visual feedback, we found fluctuations in writing pressure during the eight intervention sessions without visual feedback. In the second session, there was a decrease, followed by an increasing trend in surface pressure from the third to the sixth session. In the last two sessions, the pressure was lower than the initial pressure recorded in the pre-intervention session. Although ultimately, as expected, a decrease in pressure compared to the pre-intervention sessions was observed, there appeared to be greater variance and less consistency in the writing pressure without the visual feedback effect. These contrasting findings regarding the reliance on visual feedback among children with DCD might be explained by differences in sensory information processing, leading to a type of sensory compensation in this population [37]. A temporary increase in pressure is possibly required to amplify the additional sensory feedback necessary for pressure regulation. 

Conversely, without visual feedback, there is less control and awareness of the feedback component, resulting in more fluctuations and less consistency. A study among children with DCD and with typical development found no clear association between writing pressure and writing outcomes. However, it noted a negative correlation between increased writing pressure and the percentage of unidentified words written [37].

The various techniques and motor tasks in different studies have generated substantial variability in findings, making it challenging to draw definitive conclusions. It appears that the tendency to increase pressure during writing activity is the underlying cause of fatigue in writing tasks among children with DCD over prolonged periods of writing.

Additionally, analyses of handwriting characteristics included numerous personal features, such as letter size, shape, slant, position on the line, and more. These components vary according to each child’s personal handwriting profile. In the current study, no changes were observed in these features following the intervention, but the handwriting consistency component appeared to contribute significantly to writing legibility. Therefore, the finding that the intervention also contributed to consistency in writing pressure enhances its contribution to more fluent and legible handwriting.

### 4.4. Transferability

After the children had practiced the same text in eight intervention sessions, we observed their ability to transfer the acquisition of writing components to a similar but new task. We found significant decreases in time measures when comparing temporal, spatial, and writing pressure measures in writing the new text post-intervention to writing the pre-intervention text. In addition, there was an increase in writing pressure and a significant decrease in pressure variance but no significant differences in the spatial measures pre-intervention compared to the new text post-intervention (except in the standard deviation of spacing between letters). These findings are similar to findings comparing the pre- and post-intervention measures of writing the same text. Thus, they support the apparent transferability of learning to a new task.

These findings are also similar to studies that demonstrated learning and transfer abilities in children with DCD [16,17] and extend the findings of Adi Japha and Berstel’s study [39], where transferability of learning to a new task was not observed. In that study, acquisition focused on writing new shapes among younger children (5–8 years); our study involved a similar writing task within the scope and type of the standard handwriting assessment among a broader age range (7–11 years). However, the improvements in our study were mainly evident in temporal measures; no significant change was seen in spatial measures. It appeared that children with DCD may require more specific prompts, support, and guidance to improve learning the spatial aspects of performance, such as size, spaces, shape accuracy.

When comparing the five segments of the new text to the five pre-intervention segments, we found differences in the first two segments in the visual feedback condition, similar to the results of the practice text. Likewise, when comparing the five segments of the eighth and last intervention to the corresponding five segments in writing the new text, no significant difference was found in pressure in any segment, strengthening the conclusion on the learning and transferability observed after the intervention period. The fact that the findings in writing a new text are very similar to those in writing the practice text indicates the presence of learning and transferability. Writing practice and intervention among children with DCD can improve their academic performance even in similar writing activities for which they have not specifically trained.

An important consideration in our study design was the ability to perform within-subject analyses. Whereas similar previous studies had focused on the new acquisition process [4,44], we distinguished between the specific gains derived from the visual feedback condition and general, cumulative learning, which included training with and without visual feedback in each session. Along with examining differences within the test group (subject differences), a strength of this study lies in testing both feedback conditions with the same population, despite the lack of a control group. This approach reduced confounding variable biases related to natural development, additional learning, and other uncontrolled curriculum activities A traditional A–B–A design is more complex to implement in an educational system because it lasts longer and may involve additional resources and interferences, such as holidays. The A–B–A model is a research design in behavior analysis where a baseline measurement (A) is taken, followed by an intervention or treatment (B), and then the baseline measurement is reinstated (A) to observe changes in behavior due to the intervention. This model helps assess the effectiveness of interventions while considering potential fluctuations in behavior over time [57]. Further, studies like ours that integrate innovative technologies commonly start with more limited models to obtain preliminary findings and test the tool’s objectivity and user-friendliness [58].

Another concern was the stigma that children with DCD participating in the study might experience compared to their peers. However, it seems that using technological tools and writing on a tablet rather than on paper provided a unique experience, enhancing the children’s motivation [26]; thus, they cooperated throughout the intervention sessions.

An important component of this study is the identification and treatment of children’s writing difficulties within the school environment, which represents their daily natural setting for writing. Treating writing difficulties in the natural ecological environment rather than in a laboratory setting may contribute to the relationship between intervention, learning, and real-life implementation in the school [38]. 

Another unique aspect of this study that formed the basis of the method is implementing the activity (writing) with personal responsibility and autonomy to promote self-identity. Transferring the responsibility and task autonomy to the students themselves encourages their self-direction without depending heavily on adult (therapist, teacher, or parent) feedback. It is a suitable anchor for new learning paradigms in schools adapted to the 21st century [59]. The therapeutic process that leads the children to pay maximum attention to the quality of their performance in writing also provides them tools for independent coping in their daily academic functioning. Additionally, this method shows the potential for remote intervention with monitoring, which can shorten the waiting lists for treatment and save costs.

### 4.5. Limitations and Recommendations for Future Research

Methodological limitations should be acknowledged. First, there was no control group (that did not receive any treatment). However, a comparison within the test group was conducted between the interventions with and without computerized visual feedback, allowing insights into the learning process during the intervention period. Second, the sample size was relatively small, consisting of 27 children suspected of having DCD, therefore the generalization of the results is limited. A larger study population would provide more reliable and valid conclusions. Third, the research did not account for the comorbidity of attention deficits that could have had an impact on the participants’ learning ability during the intervention. Finally, the intervention frequency in this research was once a week for 8 weeks; a longer and more intensive intervention period might lead to more significant improvements. 

We recommend continuing to research the effectiveness of writing interventions over a longer period within the natural school environment and with methods that increase student responsibility and autonomy for improvement and progress. Other recommendations for future research and clinical implementation include examining long-term learning retention after intervention by reevaluating children after a specific period following the intervention completion. (This step was not possible in this research due to school closures during the COVID-19 pandemic). Investigating the intervention process involving additional partners, such as parents and teachers, is also crucial. It would elicit a broad ecological perspective on all participants in the child’s life who support the intervention process without compromising the principle of independent and autonomous training for the child.

Further, we recommend establishing a research team, including engineers and software professionals, that would develop the most accurate and efficient tool for analyzing the data obtained from the tablet. This collaboration might lead to the creation of a simple, user-friendly product that provides real-time feedback based on various measurements. It could significantly advance the treatment of writing difficulties in school and home environments, saving financial and human resources and focusing on each student’s personal and precise writing characteristics.

Lastly, an interesting follow-up research direction would be an intervention study for children with DCD in the prewriting stages using shape copying with visual feedback. The implications may contribute vital knowledge to promote the academic abilities of preschool children with DCD and aid their optimal integration among their peers upon entering the school system.

## 5. Conclusions

Despite the prevalence of digital tools in research, their clinical utilization remains limited. This study establishes the potential of using this tool for efficient and objective therapeutic interventions. It demonstrates the possibility of improving handwriting skills in children with pDCD through computerized visual feedback intervention. Improved handwriting speed, components, and consistency and reduced writing pressure variance were observed following the intervention.

## Figures and Tables

**Table 1:** Gender, dominant hand, and age variables of the research DCD group.

Characteristic	Count (*N* = 27)	%
Gender		
Male	17	62.97
Female	10	37.03
Dominant hand		
Right	21	77.77
Left	6	22.23
	*M*	*SD*
Age (years)	9.02	1.18

## Data Availability

The data presented in this study are available on request from the corresponding author. The data are not publicly available due to ethical restrictions.

## Supplementary Materials

The following supporting information can be downloaded at https://www.mdpi.com/article/10.3390/children10091534/s1, Figure S1: Calibration curve of WACOM-Cintiq-Pressure display. Figure S2: Pre- and post-intervention temporal measures of writing without visual feedback. Figure S3: Time writing one letter pre- and post-intervention, with and without visual feedback. Figure S4: Interaction effect of writing-time pre- and post-intervention, with and without visual feedback. Figure S5: Interaction effect of in-air time pre- and post-intervention, with and without visual feedback. Figure S6: Improvement in writing capacity pre- and post-intervention, with and without visual feedback. Figure S7: Interaction effect of standard deviation letter height, in time (pre-/post-intervention) X group (with/without visual feedback). Figure S8: Pre- and post-intervention spatial measures of writing without visual feedback. Figure S9: Interaction effect of the percentage of letters written, in time (pre-/post-intervention) X group. Figure S10: Mean writing pressure divided into five segments pre-intervention. Figure S11: Mean writing pressure divided into five segments post-intervention. Figure S12: Time, spatial, and pressure measures of writing new text after the intervention period compared to pre-intervention. Table S1: The visual feedback range according to the pressure levels on the tablet. Table S2: Means and standard deviations of the temporal, spatial, and pressure writing variables in children with DCD, with and without visual feedback pre-intervention (pre), post-intervention (post), and at transferability. Table S3: Means and standard deviations of the writing pressure in the intervention sessions with and without visual feedback. Table S4: Means and standard deviations of the pressure variables in children with DCD, writing with and without visual feedback, divided into five segments of the writing section, pre-/postintervention, compared to copying new text after the intervention period (transferability). Video S1: Writing example of color change in accordance to pressure https://youtu.be/rHjfZgtDQLw (accessed on 21 July 2023).
