# Supplementary material for: Enhancing Handwriting Performance of Children with Developmental Coordination Disorder (DCD) Using Computerized Visual Feedback
Source: Children (Basel). 2023 Sep 11;10(9):1534. doi: 10.3390/children10091534 (PMC10529407; doi:10.3390/children10091534)
Supplement: Supplementary file 1 [file children-10-01534-s001.zip › children-2549198-supplementary.pdf]

Supplementary Materials

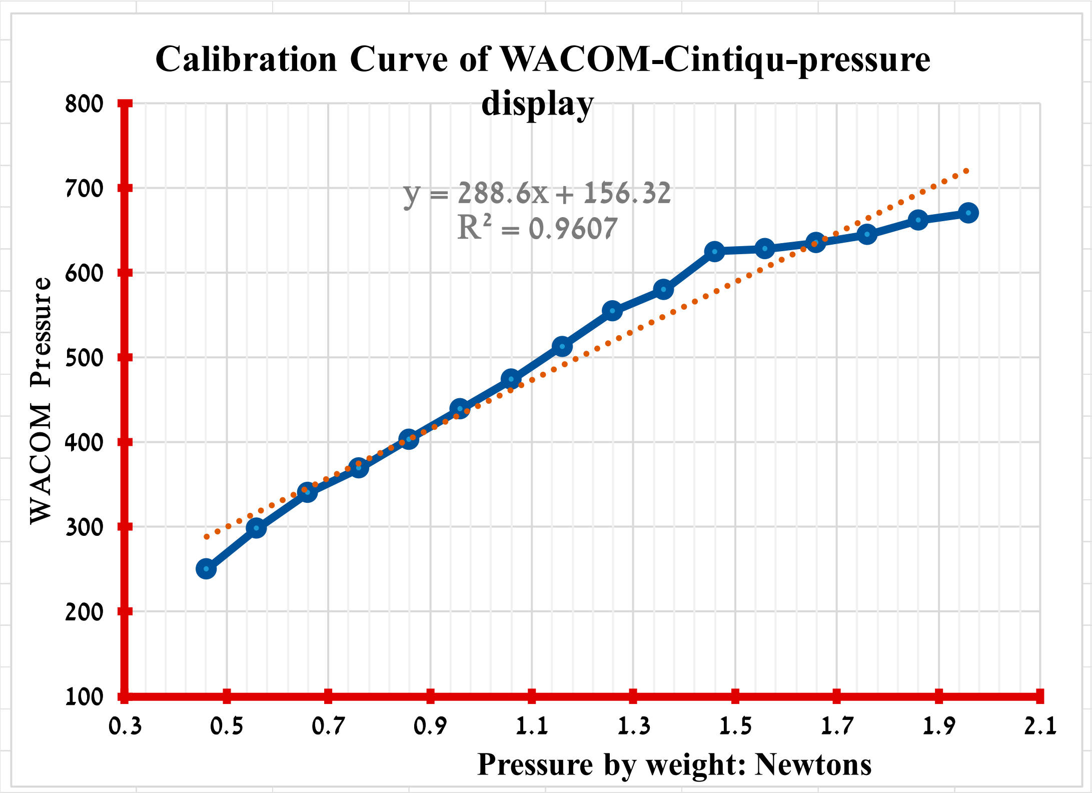

Figure S1. Calibration curve of WACOM-Cintiq-Pressure display.

Note: This is the calibration curve of the WACOM-13HD Cintiq tablet pressure display responding to the stylus used in the study. The pressure data (converted to Newtons) are the means of the three successive measures.

Color	Pressure range[a]	Meaning of the feedback
Blue	0-240	Weak pressure
Blue to black	240-540	Weak to normal pressure
Black	540-620	Norm - the degree of expected pressure
Black to red	620-920	Normal to strong pressure
Red	920-1024	Strong pressure

Table S1. The visual feedback range, according to the pressure levels on the tablet.
[a]The Wacom tablet measures a total of 0 to 1,024 degrees of pressure non-scaled.

Measure	With visual feedback					Without visual feedback		Main effect (time[a])		Group effect[b]		Interaction effect	
	Pre	Post	Transferability[c]			Pre	Post	*df*(1,26)		*df*(1,26)		*df*(1,26)	
		M (*SD*)		*F*(2,24)	η^2	*M* (*SD*)		*F*(1,26)	η^2	*F*(1,26)	η^2	*F*(1,26)	η^2
Temporal (s)													
Time all	272.08 (34.28)	213.92 (60.54)	229.38 (57.82)	12.46***	.509	243.06 (46.84)	223.56 (69.05)	27.7***	.517	4.74*	.154	22.50***	.462
One letter time	0.0059 (0.00457)	0.0035 (0.0021)	0.0036 (0.0019)	8.32**	.410	0.0040 (0.0024)	0.0039 (0.0024)	8.53**	.154	9.59**	.270	14.13**	.350
In-air time	174.33 (35.35)	131.40 (46.55)	151.31 (51.80)	4.09*	.254	160.02 (36.52)	143.23 (62.53)	17.75***	.410	0.08	-	10.85**	.290
Writing time	97.75 (27.82)	82.51 (24.58)	78.06 (19.00)	10.98**	.478	83.03 (2365)	80.33 (25.39)	8.14**	.240	10.49**	.290	5.32*	.170
Capacity	0.55 (0.18)	0.81 (0.25)	0.72 (0.23)	21.26***	.639	0.67 (0.20)	0.78 (0.29)	5.47*	.174	46.76***	.643	46.52***	.640
Spatial (mm)													
Height	34.39 (11.17)	32.52 (8.88)	31.21 (8.57)	1.78	-	33.71 (10.54)	32.66 (9.02)	1.56	-	0.53	-	1.50	-
Width	20.93 (5.55)	20.23 (4.99)	19.04 (4.26)	2.43	-	20.86 (5.40)	20.02 (5.21)	1.33	=	0.59	-	0.22	-
SD height	13.05 (4.70)	12.36 (2.59)	12.46 (2.43)	0.27	-	12.79 (4.44)	13.30 (3.15)	0.26	-	1.12	-	4.67*	.153
SD width	11.09 (2.82)	10.82 (2.31)	10.40 (2.27)	0.96	-	11.08 (2.56)	10.94 (2.57)	0.39	-	0.07	-	0.18	-
Letter space	30.43 (7.23)	30.11 (5.76)	31.66 (6.34)	3.27*	.214	29.30 (7.01)	31.45 (6.66)	0.96	-	0.94	-	7.11*	.215
Word space	70.03 (26.94)	68.62 (19.71)	72.01 (23.81)	2.09	-	67.13 (26.32)	78.95 (30.73)	1.84	-	1.56	-	3.36	-

SD letter space	25.72 (13.43)	24.58 (9.15)	28.51 (9.37)	4.79*	.285	22.84 (0.06)	27.24 (9.13)	0.95	-	0.00	-	4.91*	.215
SD word space	54.40 (37.82)	41.81 (27.70)	46.26 (34.64)	0.20	-	54.43 (42.08)	61.59 (50.96)	0.04	-	4.68*	.153	1.55	-
Letter area	493.58 (304.00	434.61 (217.68)	396.11 (183.92)	2.07	-	469.34 (227.81)	433.77 (227.42)	2.14	-	1.24	-	1.47	-
Word area	5060.91 (2580.55)	4574.02 (2103.22)	4309.26 (1881.79)	1.76	-	4822.14 (2307.80)	4706.22 (2373.42)	0.89	-	0.24	-	3.74	-
Erasures (number)	3.26 (2.55)	2.85 (1.91)	3.00 (2.46)	0.24	-	3.37 (1.84)	3.11 (2.24)	0.88	-	0.52	-	0.84	-
Letters not omitted (%)	84.94 (18.41)	93.93 (9.72)	90.19 (10.71)	7.27**	.378	90.57 (13.31)	90.62 (13.51)	8.13**	.238	1.66	_	11.45**	.306
Pressure													
Total	440.27 (65.43)	467.28 (46.31)	470.84* (47.06)	5.86**	.328	422.54 (90.74)	408.18 (80.18)	0.28	-	15.76**	.380	13.17*	.340
SD total	86.20 (13.83)	75.82 (11.37)	76.43* (11.71)	9.24**	.435	79.22 (18.14)	74.08 (15.51)	12.57**	.320	3.89	-	2.37	-

Table S2. Means and standard deviations of the temporal, spatial, and pressure writing variables in children with DCD, with and without visual feedback preintervention (Pre), postintervention (Post), and at transferability. (N = 27).

[a]Time is Pre or Post (intervention);[b]group interaction indicates intervention With or Without visual feedback; [c]transferability indicates follow-up, writing a new text after the intervention period. *$p < 0.05$; **$p < 0.01$; ***$p < 0.001$.

Additional Temporal Measures

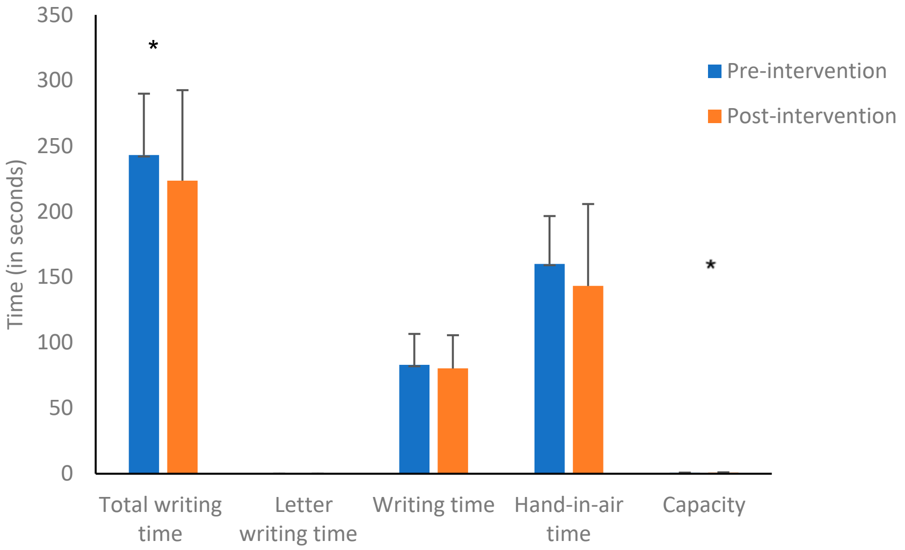

Figure S2. Pre- and postintervention temporal measures of writing without visual feedback. In writing without visual feedback postintervention, there was a significant decrease only in the time to copy the whole section. **p* < 0.05; ***p* < 0.01; ****p* < 0.001.

Comparing the time for writing one letter revealed a significant effect on the measurement time, F(1,26) = 8.53, p = .007, $\eta^2 = .247$. The duration of writing one letter preintervention was significantly longer than at postintervention. Additionally, a significant effect of intervention condition (with or without visual feedback) was found, F(1,26) = 9.59, p = .005, $\eta^2 = .270$, indicating that writing one letter with visual feedback was slightly longer than without visual feedback. Furthermore, a significant interaction between time and intervention condition was found, F (1,26) = 14.13, p = .001, $\eta^2 = .35$, (Figure S3).

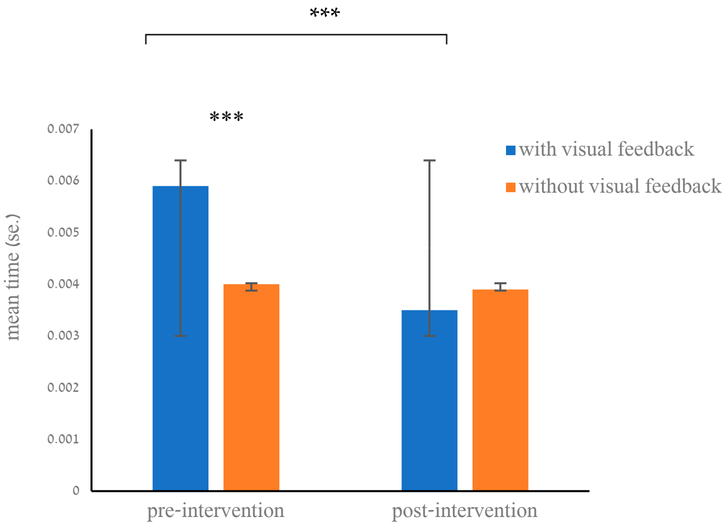

Figure S3. Time writing one letter pre- and postintervention, with and without visual feedback. **p* < 0.05; ***p* < 0.01; ****p* < 0.001.

Post hoc analyses (*t* tests) were conducted to examine the source of the interaction. When the children received visual feedback preintervention, their duration to write one letter was slightly longer (*M* = 0.0059, *SD* = 0.0047) compared to writing one letter without visual feedback

(M = 0.0040, SD = 0.0024), $p \leq 0.001$. In contrast, no significant differences were found postintervention between performance with and without visual feedback. Further analysis of the source of the interaction indicated a significant reduction in the average duration of writing one letter with visual feedback compared to postintervention, $p \leq 0.001$. In contrast, the duration of writing one letter without visual feedback did not significantly decrease.

In examining the writing-time (time during which the pen touches the surface, excluding the in-air time), a significant effect of time (pre-/postintervention) was found, $F(1,26) = 8.14$, $p = .008$, $\eta^2 = .24$. The preintervention writing time (with visual feedback: $M = 97.75$, $SD = 27.82$; without visual feedback: $M = 83.03$, $SD = 23.65$) was longer than the postintervention writing time (with visual feedback: M = 82.51, SD = 24.58; without visual feedback: M = 80.33, $SD = 25.39$). Additionally, a significant effect of intervention condition (with or without visual feedback) was found, $F(1,26) = 10.49$, $p = .003$, $\eta^2 = .29$, as was a significant interaction between time and condition, $F(1,26) = 5.32$, $p = .03$, $\eta^2 = .17$, (Figure S4).

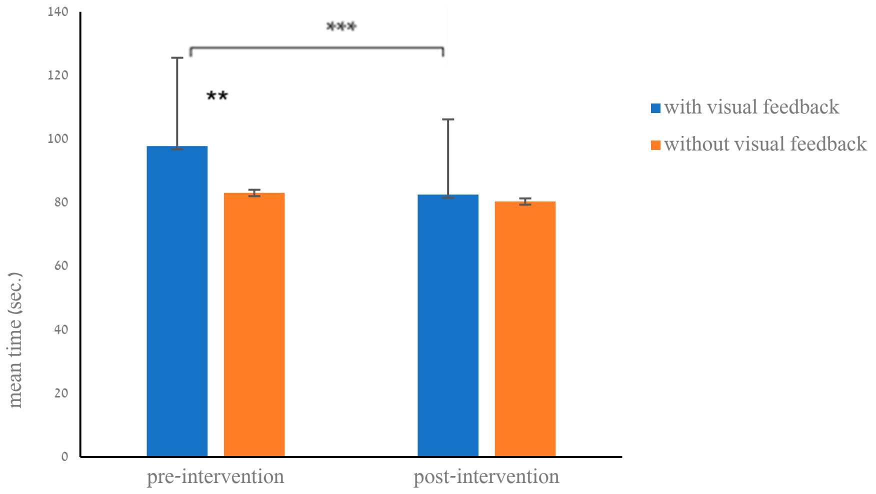

Figure S4. Interaction effect of writing-time pre- and postintervention, with and without visual feedback. *$p < 0.05$; **$p < 0.01$; ***$p < 0.001$.

Post-hoc analyses indicated that when children received visual feedback preintervention, the writing time was significantly longer compared to the writing time without visual feedback, $p \leq 0.001$. However, postintervention, no significant differences were found between the writing time with and without visual feedback, but the performance duration of in both conditions decreased. Another examination of the interaction's source showed that the writing time with visual feedback significantly decreased from preintervention to postintervention, $p = 0.002$. In contrast, the writing time without visual feedback did not significantly decrease.

Upon examining the in-air time (i.e., the time between writing letters or between words), a significant effect of time (preintervention compared to postintervention) was found, $F(1,26) = 17.75$, p = 0.000, η^2 = 0.41. The in-air time was longer at preintervention than at postintervention. However, no significant effect of intervention condition was found. In addition, a significant interaction of time X intervention condition was found, $F(1,26)= 10.85$, $p = 0.003$, $\eta^2 = 0.29$ (Figure S5). Post-hoc analyses (t tests) were conducted to examine the source of the interaction. The analysis of preintervention differences indicated that with visual feedback, the in-air time was longer than without visual feedback, p = .005. However, postintervention, no significant differences were found, the in-air time decreased in both conditions. Further examination of the interaction source revealed that the in-air time with visual feedback was significantly shorter preintervention than postintervention, $p < 0.001$. In contrast, the in-air time without visual feedback showed no significant decrease.

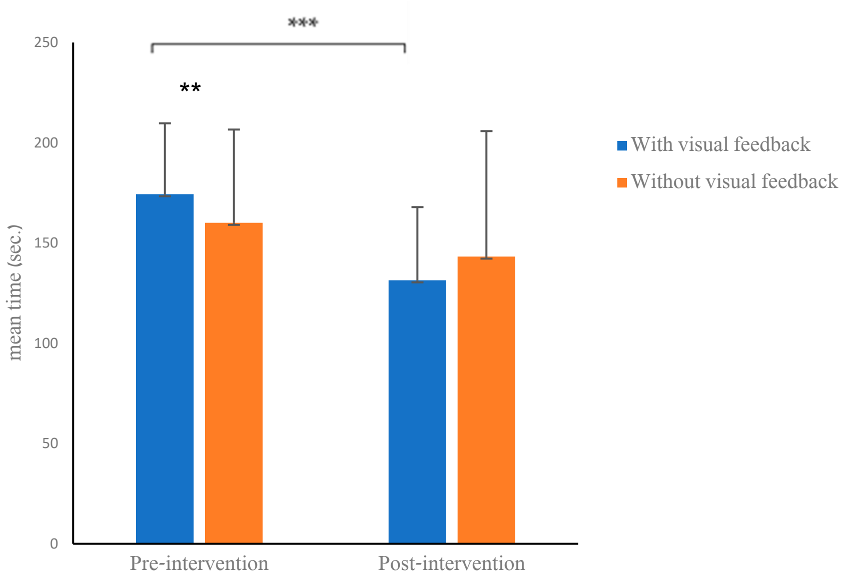

Figure S5. Interaction effect of in-air time pre- and postintervention, with and without visual feedback. *$p < 0.05$; **$p < 0.01$; ***$p < 0.001$.

When examining the writing capacity (number of letters written in the total writing time) postintervention compared to pre-intervention, findings showed a significant main effect for time (pre-/postintervention), $F(1,26) = 5.47$, $p = .03$, $\eta^2 = .174$. A significant effect for intervention condition was found, $F(1,26) = 46.76$, $p = .000$, $\eta^2 = .643$. Figure S6 shows that the writing capacity with visual feedback was significantly higher than the writing capacity without feedback. Furthermore, a significant interaction effect of time (pre-/postintervention) X intervention condition was found, $F(1,26) = 46.52$, $p = .000$, $\eta^2 = .64$, (Figure S6).

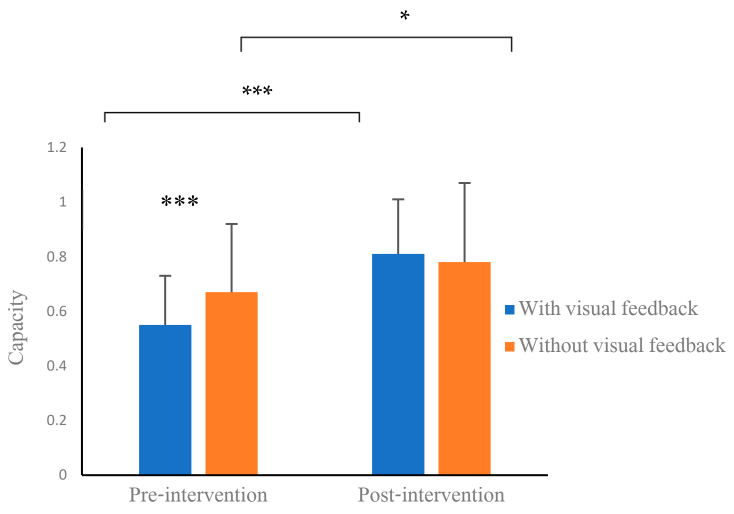

Figure S6. Improvement in writing capacity pre- and postintervention, with and without visual feedback. *$p < 0.05$; **$p < 0.01$; ***$p < 0.001$.

Post hoc analyses (*t* tests) were also conducted to examine the source of the interaction. The analysis of differences indicated that, preintervention, the writing capacity was lower when the children received visual feedback (M = 0.55, SD = 0.18) than when they did not

($M = 0.67$, $SD = 0.20$), $p < 0.001$. However, no significant differences were found between the postintervention writing capacity with ($M = 0.81$, $SD = 0.25$) and without visual feedback ($M = 0.78$, $SD = 0.29$). Furthermore, an additional examination of the source of the interaction revealed that the preintervention writing capacity with visual feedback significantly increased compared to the postintervention writing capacity, $p \leq 0.001$, as did the writing capacity without visual feedback, $p = 0.03$.

Additional Spatial Measures

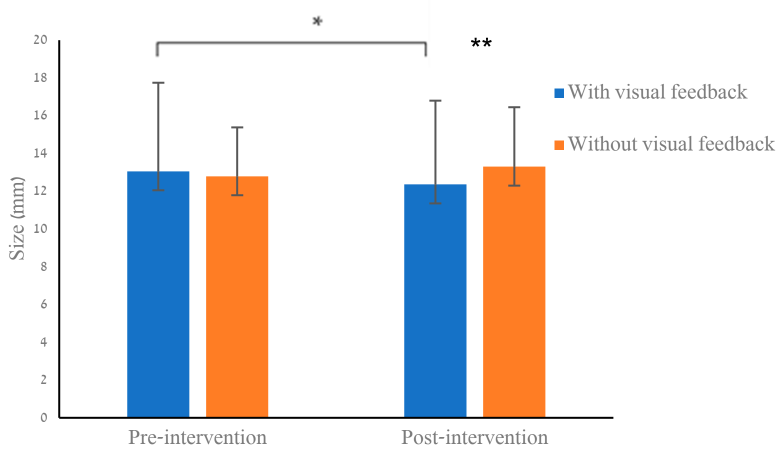

Figure S7. Interaction effect of standard deviation letter height, in time (pre-/postintervention) X group (with/without visual feedback). *$p < 0.05$; **$p < 0.01$; ***$p < 0.001$.

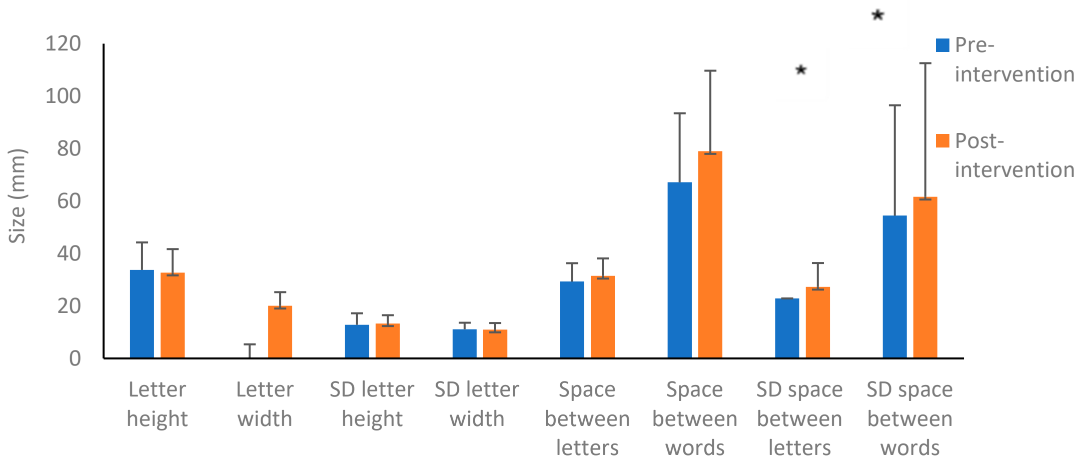

Figure S8. Pre- and postintervention spatial measures of writing without visual feedback. *$p < 0.05$; **$p < 0.01$; ***$p < 0.001$. A significant increase was found in the standard deviation of the space between letters and between words.

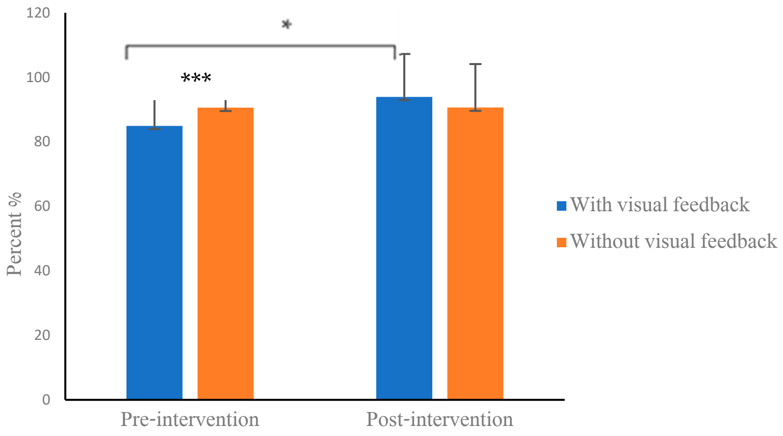

Figure S9. Interaction effect of the percentage of letters written, in time (pre-/postintervention) X group. *$p < 0.05$; **$p < 0.01$; ***$p < 0.001$.

Upon examining additional spatial variables, such as the mean height and width of the letters, no significant differences were found for the effect of time (pre- or postintervention) and intervention condition type (with/without visual feedback) on the height and width of the letters. There also was no significant interaction effect, nor were significant differences found in the spacing between letters and words, the letter and word area, or the number of erasures and corrections between pre- and postintervention.

Because the mean of spacing between letters and words does not necessarily reflect actual changes due to compensation between large and small spacings, we also examined the standard deviations. We found no significant differences in the standard deviations of the mean spacing between letters and between words in the intervention with visual feedback when comparing pre- and postintervention. However, we found significant differences in the standard deviations of the mean spacing between letters in the without feedback condition preintervention ($M = 22.84$, $SD = 0.06$) and postintervention ($M = 27.24$, $SD = 9.13$), $p = .007$.

An examination of the standard deviations of the mean spacing between words revealed no significant difference for time (pre-/postintervention), a significant difference for intervention condition, $F(1,26) = 4.68$, $p = .04$, $\eta^2 = .153$, and no interaction effect. The standard deviation of the spacing between words increased postintervention in the intervention without visual feedback and decreased slightly (but not significantly) with feedback. It can be concluded that there was a significant increase in the standard deviation of the spacing between letters and between words in the intervention condition without visual feedback.

Additional Pressure Measures

After comparing the overall writing pressure preintervention to postintervention, we made another comparison to examine the differences in the writing pressure across all eight sessions of intervention period (Figure 7).The findings indicated a significant pressure decrease only in the without visual feedback condition between the sixth (M = 443.30, SD = 81.96) and seventh (M = 407.65, SD = 71.83) sessions, $F(1,25) = 3.37$, $p = .01$, $\eta^2 = .554$. In the visual feedback condition, no significant differences were found in the writing pressure across any of the eight sessions (Table S3).

Measure	With visual feedback		Without visual feedback	
Session	M	SD	M	SD
1	441.72	66.28	429.22	85.05
2	445.03	44.60	427.50	69.26
3	458.02	46.09	399.93	84.24
4	450.10	51.37	405.65	87.25
5	461.67	42.55	422.85	111.85
6	460.93	52.15	443.30	81.96
7	462.73	39.96	407.65	71.83
8	466.86	47.18	408.88	81.68

Table S3. Means and standard deviations of the writing pressure in the intervention sessions with and without visual feedback.

Writing Pressure Divided into Five Segments

To examine whether the children maintained the same pressure level throughout the writing task, we divided the writing task into five equal segments (by the number of letters the child wrote in the passage); see Table S4).

Measure	With visual feedback		Without visual feedback		Transferability
Pressure segment	Preintervention *M* (*SD*)	Postintervention *M* (*SD*)	Preintervention *M* (*SD*)	Postintervention *M* (*SD*)	*M* (*SD*)
1	408.21 (69.42)	464.01*** (44.78)	415.48 (83.13)	406.70 (83.62)	479.06 (44.36)
2	428.19 (81.70)	463.02* (43.08)	409.77 (93.78)	395.72 (78.90)	467.19 (45.25)
3	442.23 (75.35)	470.12* (48.76)	419.56 (100.77)	410.07 (80.15)	469.33 (54.65)
4	458.80 (68.84)	471.90 (53.75)	426.89 (96.00)	415.22 (85.85)	479.65 (55.64)
5	462.63 (65.09)	467.38 (57.09)	439.72 (97.91)	412.98 (89.06)	459.55 (53.10)

Table S4. Means and standard deviations of the pressure variables in children with DCD, writing with and without visual feedback, divided into five segments of the writing section, pre-/postintervention, compared to copying new text after the intervention period (transferability). *p < 0.05; **p < 0.01; ***p < 0.001.

Preintervention, significant differences were observed in the visual feedback condition between Segment 1 ($M = 408.21$, $SD = 69.42$) and Segments 3 ($M = 442.23$, $SD = 75.35$, $p = 0.006$); 4 ($M = 458.80$, $SD = 68.84$, $p = 0.001$) and 5 ($M = 462.63$, $SD = 65.09$, $p = 0.001$) but none in the without visual feedback condition (Figure S10).

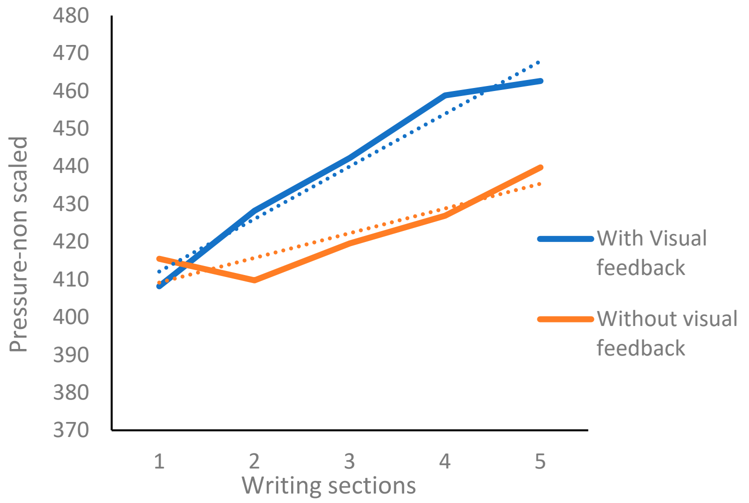

Figure S10. Mean writing pressure divided into five segments preintervention.

Postintervention, no significant differences were found between the five segments in the writing pressure for either condition, with or without visual feedback (Figure S11).

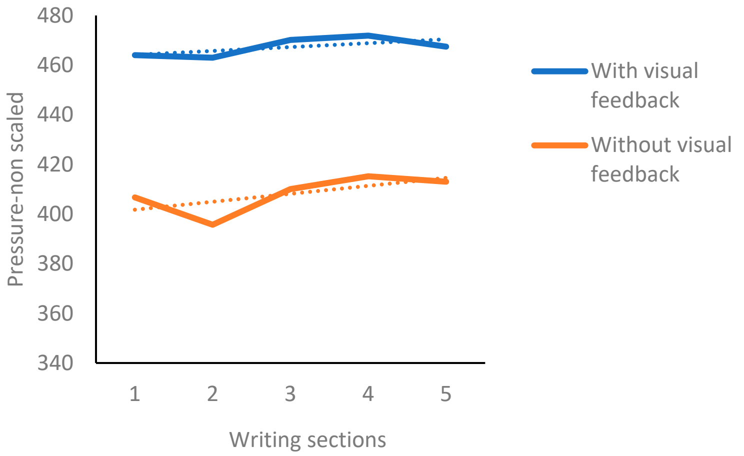

Figure S11. Mean writing pressure divided into five segments postintervention.

In *t* tests comparing the performance of each pre- and postintervention segment, significant differences were found in the visual feedback condition between Segment 1 preintervention ($M = 408.21$, $SD = 69.42$); postintervention ($M = 464.01$, $SD = 44.78$), $t(26) = 4.54$, $p = .000$; Segment 2 preintervention ($M = 428.19$, $SD = 81.70$); postintervention ($M = 463.02$, $SD = 43.08$), $t(26) = 2.46$, $p = .021$; and Segment 3 preintervention ($M = 442.23$, $SD = 75.35$); postintervention ($M = 470.12$, $SD = 48.76$), $t(26) = 2.32$, $p = .028$. No significant differences were found in the comparisons between different segments in the without visual feedback condition.

Additional Transferability Measures (New Text)

Additional significant differences were found when comparing the writing pressure between the five segments of the new text, $F(4,22) = 6.57$, $p = .001$, $\eta^2 = .545$. A significant difference was observed between the fourth ($M = 479.65$, $SD = 55.64$) and fifth segment ($M = 459.55$, $SD = 53.10$), $p = 0.003$, indicating a decrease in pressure toward the end of the new writing text.

We compared the five segments of the new writing text to their corresponding segments in the first preintervention session in the without visual feedback condition (matching each segment to its parallel segment). The average writing pressure in the first four segments of the new text was significantly higher, respectively, $t(25) = 3.57$, $p = .001$; $t(25) = 2.78$, $p = .01$; $t(25) = 3.83$, p=.001; and $t(25) = 4.36$, $p = .000$. In the with visual feedback condition, the writing pressure in the first two segments of the new text was significantly higher compared to the writing pressure in the first preintervention session, respectively, $t(25) = 2.41$, $p = .023$, and $t(25) = 4.86$, $p = .000$.

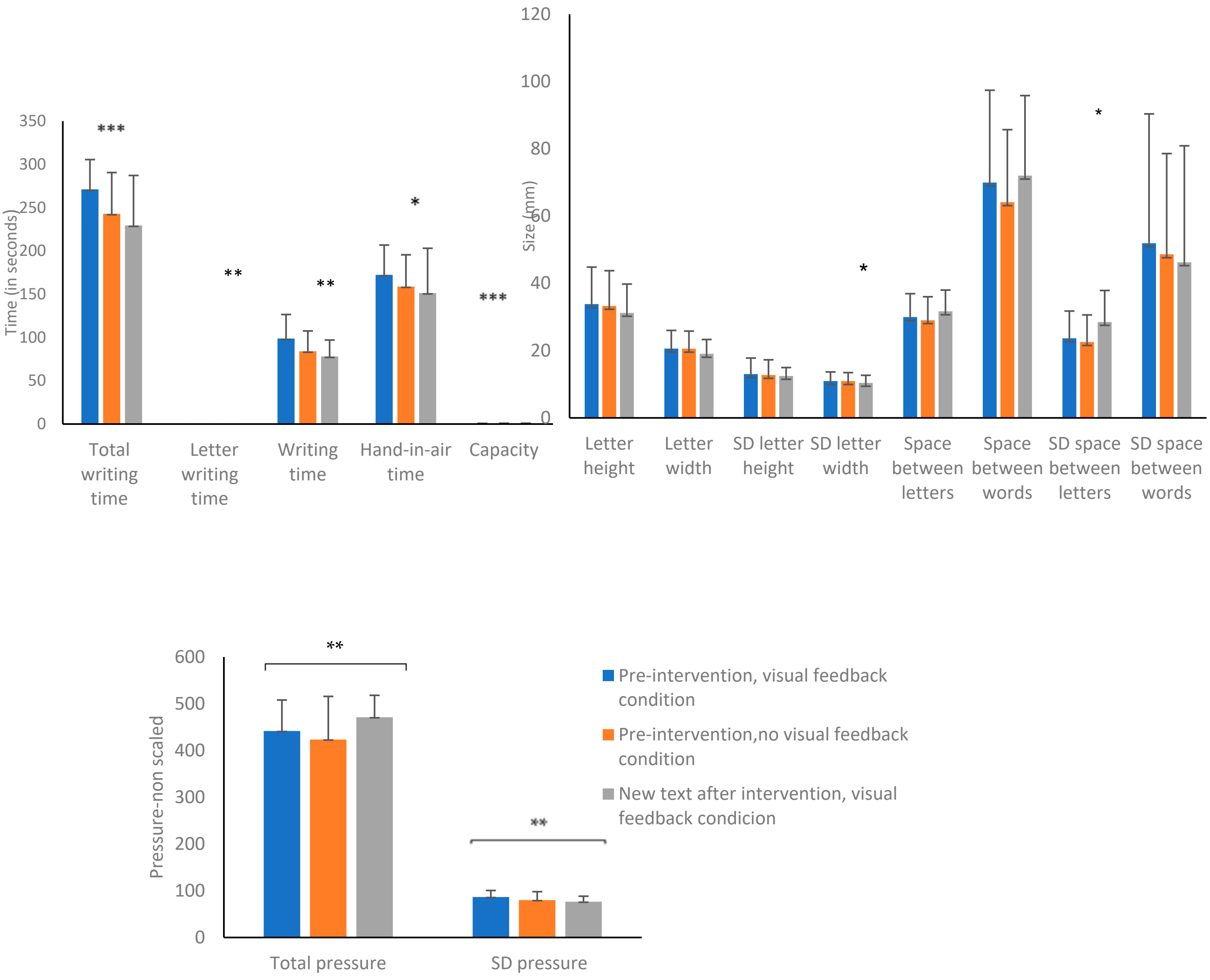

Figure S12. Time, spatial, and pressure measures of the writing new text after the intervention period compared to preintervention. *$p < 0.05$; **$p < 0.01$; ***$p < 0.001$.
